# Solitary vaginal paraganglioma with mature sacrococcygeal teratoma: a rare case report

**DOI:** 10.1186/s12902-021-00806-6

**Published:** 2021-07-07

**Authors:** Zhan Wang, Hua Fan, Jinghua Fan, Samuel Seery, Wenda Wang, Yushi Zhang

**Affiliations:** 1 Department of Urology, Peking Union Medical College Hospital, Chinese Academy of Medical Science and Peking Union Medical College, 100730 Beijing, China; 2 Department of Pathology, Peking Union Medical College Hospital, Chinese Academy of Medical Science and Peking Union Medical College, 100730 Beijing, China; 3 School of Humanities and Social Sciences, Chinese Academy of Medical Sciences and Peking Union Medical College, 100730 Beijing, China; 4 Faculty of Health and Medicine, Division of Health Research, Lancaster University, Lancaster, LA1 4YW UK

**Keywords:** Vaginal paraganglioma, Neuroendocrine tumor, Mature sacrococcygeal, Teratoma, Case report

## Abstract

**Background:**

Vaginal paraganglioma are rare, atypical, solitary tumors which originate from the female genital tract. Sacrococcygeal teratoma are also rare neoplasms which derive from one (or more) primordial germ cell layers. Here we report a unique case of vaginal paraganglioma with sacrococcygeal teratoma.

**Case presentation:**

A 44-year-old female experienced paroxysmal hypertension, palpitations and dizziness for almost six years. Enhanced CT and MRI highlighted two abnormal soft tissue lesions located in the left vaginal wall and coccyx anteriorly, and Iodine-131 metaiodobenzylguanidine (^131^I-MIBG) demonstrated abnormal radioactive uptake in perineum area. Endocrine tests showed elevated plasma normetanephrine (NMN) and 24 h urine norepinephrine. There was a well-circumscribed soft tissue lesion of approximately 3.5 cm in the left lateral vaginal wall which could be palpated during bimanual examination, together with a 1.5 cm tumor in the posterior wall of the rectum. We completely resected the two lesions in stages with the support of a senior gynecologist and general surgeons. Postoperative histopathological examinations suggested the vaginal paraganglioma and mature sacrococcygeal teratoma. Targeted sanger sequencing for the 36 mostly common paraganglioma-related genes, with a depth of 1000x, revealed no mutations. Post-operatively, plasma NMN and 24 h urine norepinephrine returned to the normal range and her symptoms completely disappeared.

**Conclusions:**

We reported an extremely rare case and the successful treatment of functional vaginal paraganglioma coexisting with adult sacrococcygeal teratoma.

**Supplementary Information:**

The online version contains supplementary material available at 10.1186/s12902-021-00806-6.

## Background

As a rare neuroendocrine tumor, paraganglioma (PG) mainly originates from neural crest cells related to the autonomic nervous system [1]. The distribution of PG is determined according to paraganglia location, ranging from the cranial base to the prostate. However, there are extremely rare cases where PG are located in the vaginal wall. There are only 10 previously reported cases to be found within the literature. Derived from the primordial germ cells and located behind the rectum, adult sacrococcygeal teratoma (SCTs) are also extremely rare with a rate of 1:40,000 to 63,000 [2]. Although most SCTs are benign, the tumor may also become malignant with an incidence up to 12 % [3]. Herein, we report a peculiar case of a patient who developed functional vaginal PG and SCT simultaneously. This is the first report of such case.

## Case presentation

A 44-year-old female presented with an history of paroxysmal hypertension, dizziness and palpitations for more than six years. A vaginal tumor had been found approximately 9 months prior to presentation. Over the past six years, the patient experienced paroxysmal hypertension without definite inducement and the blood pressure could even reach 240–250 mmHg (systolic blood pressure) and 110–120 mmHg (diastolic blood pressure). These were accompanied by bouts of dizziness, palpitations, frequent nausea and vomiting. Each attack lasted approximately 10 min but subsided of their own accord. On occasion, during sexual intercourse or defecation, the aforementioned symptoms also appeared. During bimanual examination, a well-circumscribed lesion on the left vaginal wall could be palpated, at which point the patient experienced simultaneous transient hypertension with a peak systolic of 240 mmHg and diastolic of 120 mmHg. Another soft, well-defined lesion was also found on the posterior wall of the rectum. The patient denied the similar symptoms or relative neuroendocrine neoplasms in her immediate family members.

**Fig. 1:**
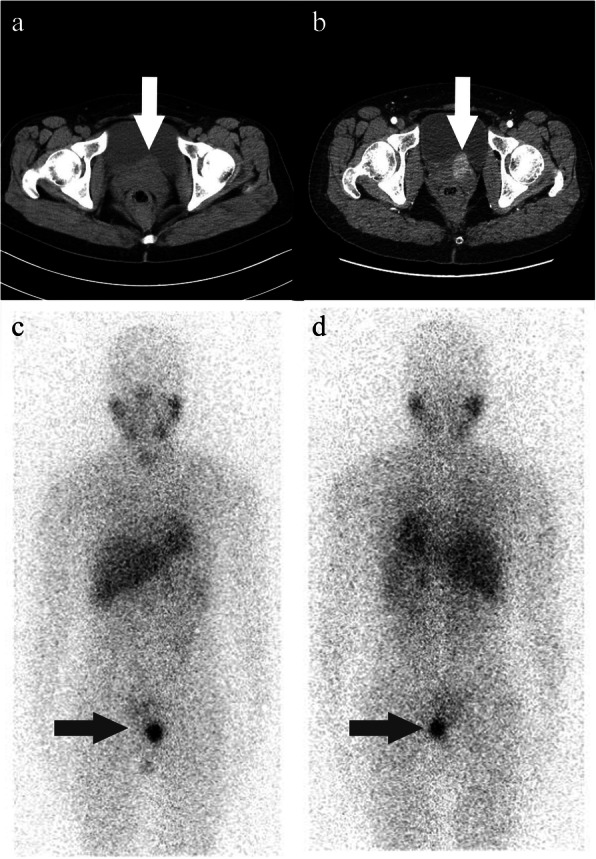
CT and ^131^I-MIBG findings of vaginal paraganglioma. **a**: abdominal and pelvic CT scan showed a mass (white arrow) on the left side of the vagina, protruding into the bladder; **b**: enhanced scan showed obvious enhancement of the tumor (white arrow); **c** and **d**: ^131^I-MIBG scan (anteroposterior and posteroanterior position respectively) showed abnormal increase of radioactive uptake on the left side of perineum area (black arrow)

**Fig. 2:**
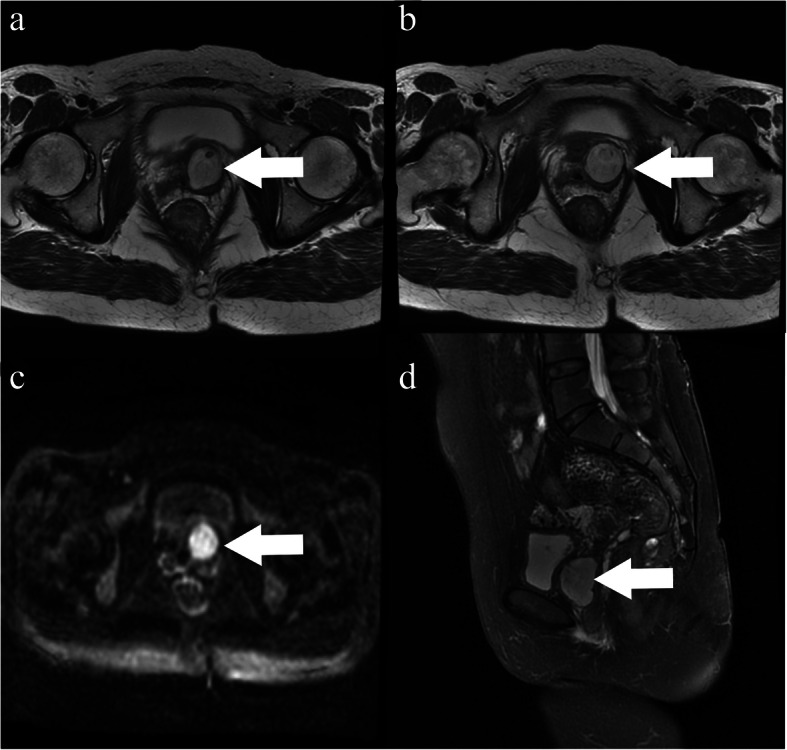
MRI findings of vaginal paraganglioma. **a** and **b**: T2WI showed high signal on the left side of the vagina (white arrow); **c**: the lesion showed high signal on DWI (white arrow); **d**: T2WI fat suppression phase (sagittal position) showed that the tumor (white arrow) was located behind the bladder, and closely related to the rectum

**Fig. 3:**
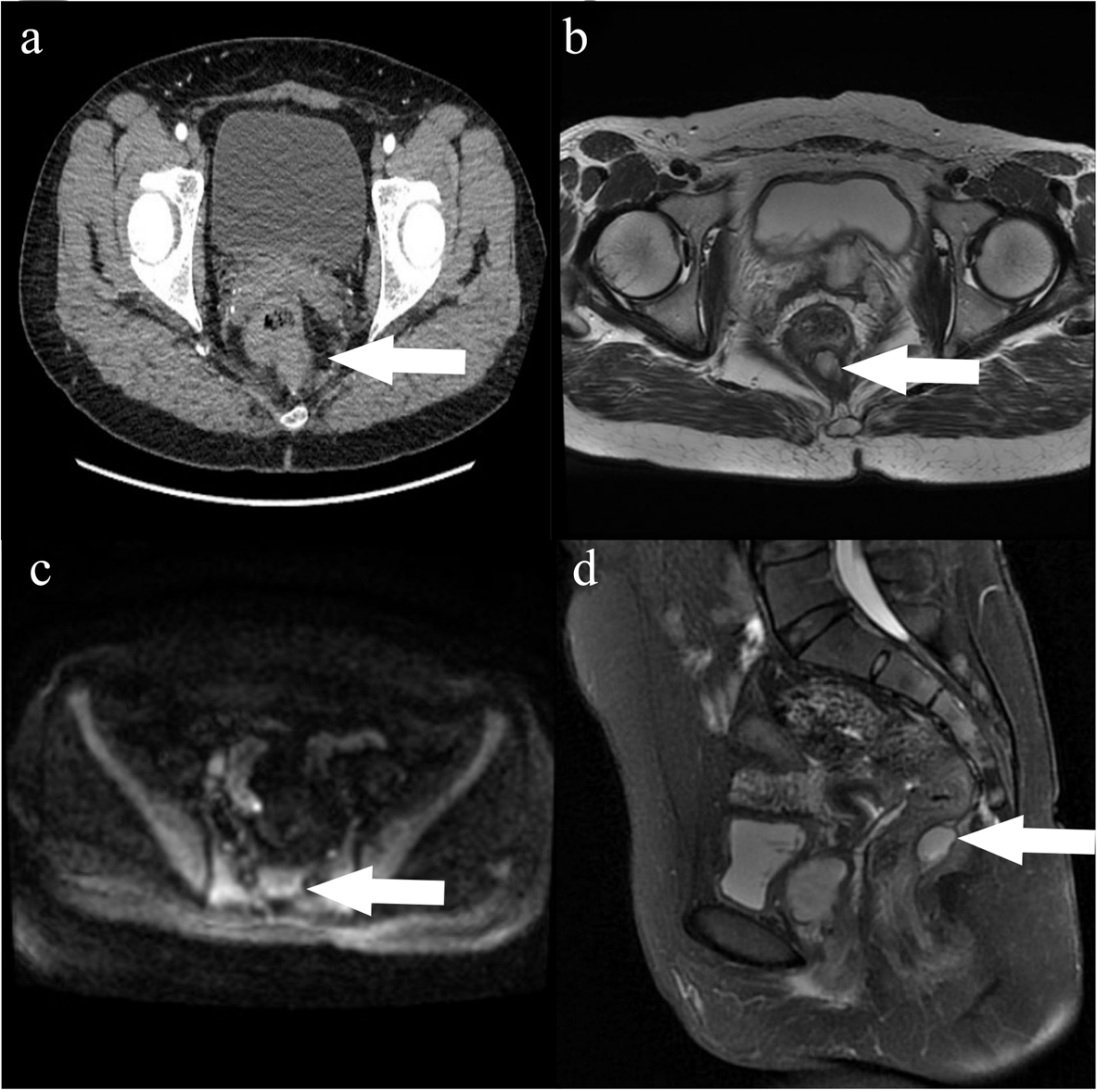
The imaging of sacrococcygeal teratoma. **a** and **b**: CT and MRI imaging demonstrated that the lesion (white arrow) located behind the rectum and in front of the coccyx. **c**: the lesion showed high signal on DWI (white arrow); **d**: T2WI fat suppression phase (sagittal position) showed that the tumor was located behind the rectum (white arrow)

Abdominal CT scans revealed a soft-tissue density shadow measuring 3.2 cm, with obvious enhancement on the left vaginal wall (Please see Fig. 1A and B) and rectum posteriorly (Please see Fig. 3A for pertinent scans). Enhanced MRI also identified two abnormal signal neoplasms located in the vagina (Please see Fig. 2A-D) and behind the rectum (As Fig. 3B-D indicated), which appeared to be in close proximity to the tailbone (please see Fig. 3D). ^131^I-MIBG examinations revealed an obvious contractive accumulation in the perineum area (Fig. 1C and D). Endocrine tests highlighted an elevated plasma normetanephrine (NMN) level of 4.70 nmol/L (reference < 0.9nmol/L), and a 24 h urine norepinephrine (NE) of 78.32ug/24 h (reference < 40.65ug/24 h). Based upon these findings, the patient was diagnosed with paraganglioma and preoperative preparations were conducted including the administration of phenoxybenzamine 5 mg q8h, absorption of fluid and implantation of bilateral DJ tubes to protect the patient’s ureters.

**Fig. 4:**
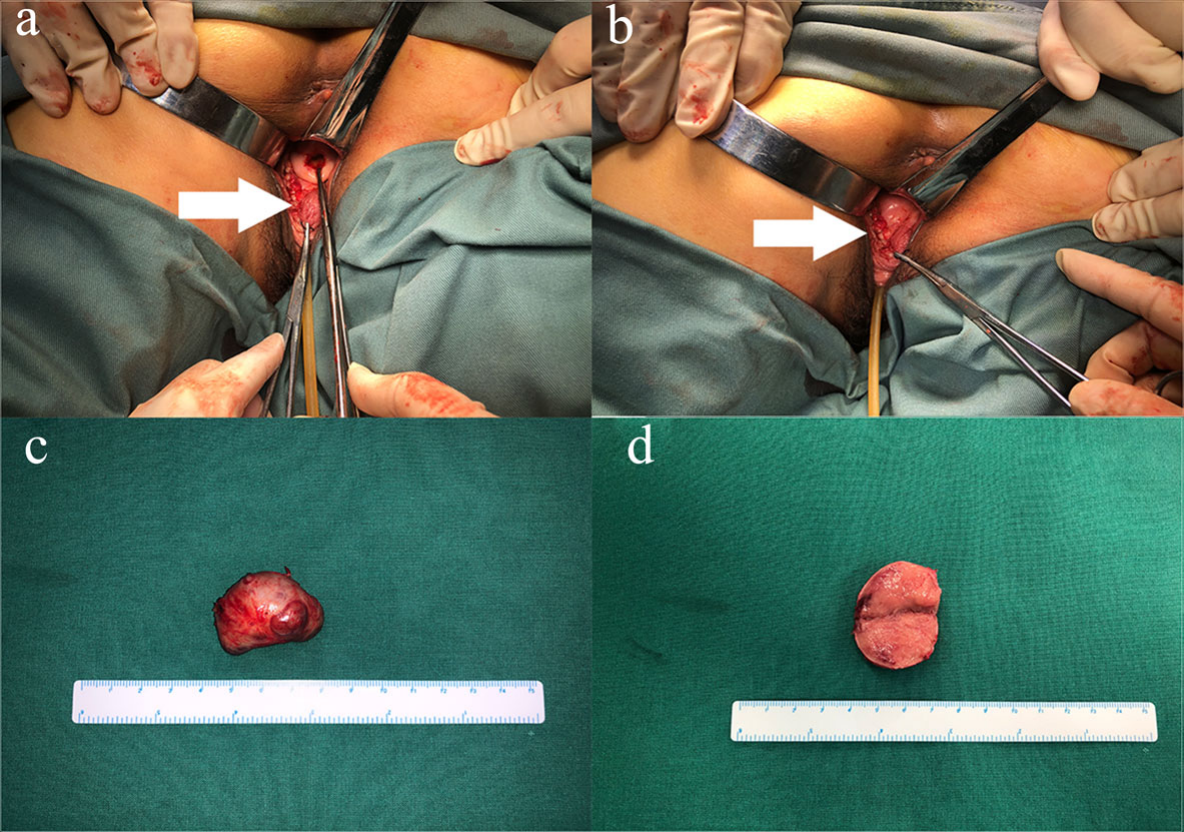
Exploration of vaginal tumor during operation and resected specimen. **a**: the image before operation (white arrow); **b**: the image after operation (white arrow); **c**: general image of the tumor; **d**: general images of the incised tumor

Through shared-decision making the patient opted for vaginal lesion resection, under direct visualization. Since the tumor located so close to the rectum and vagina, we consulted both general surgeons and a gynecologist in order to avoid damaging rectum and for vaginal reconstruction. The tumor was then successfully removed without any complications and the blood pressure during the process was quite stable. The tumor was a golden, soft but well-defined nodule measuring approximately 3.5 cm. An image has been provided, please see Fig. 4. After the operation, plasma NMN and 24 h urinary NE returned to the normal range on the second day and the symptoms ceased to occur thereafter. One week later, the patient underwent a second operation to remove the posterior rectum lesion with the assistance of general surgeons. Contrary to anticipation, the lesion appeared like a small cyst containing gelatinous substance.

**Fig. 5:**
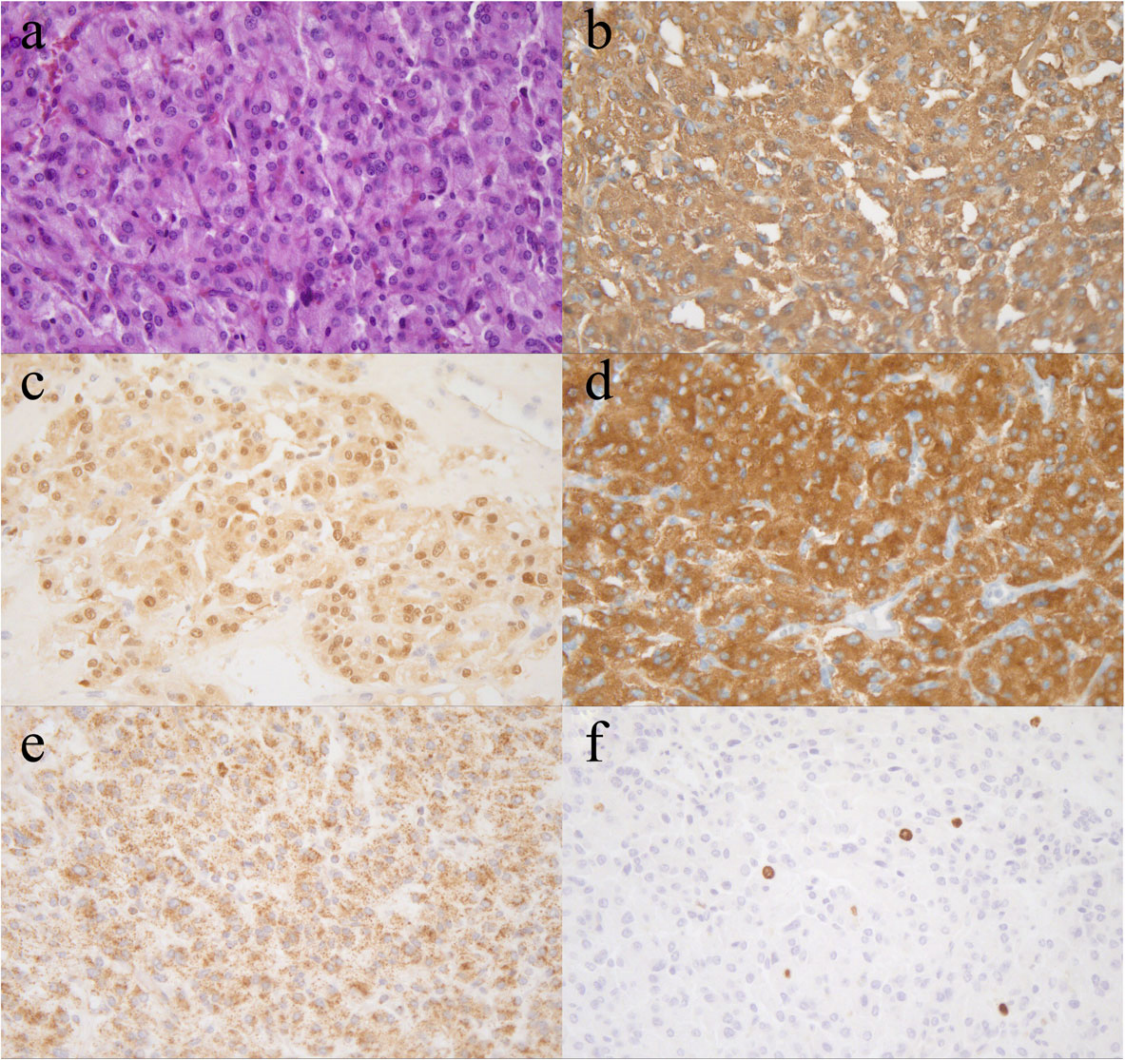
The HE staining and immunohistochemical results of the vaginal tumor (400×). **a**: HE staining; **b**: CgA (+); **c**: S100 (+); **d**: Syn (+); **e**: SDHB (+); **f**: Ki-67 rate (3%)

**Fig. 6:**
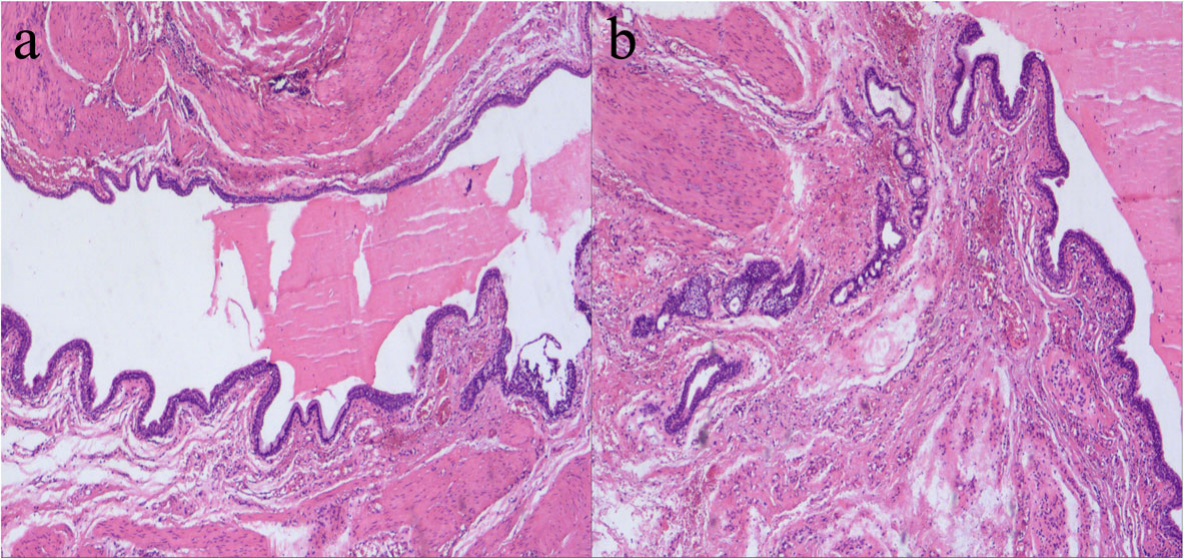
The HE staining of the sacrococcygeal teratoma. **a** and **b**: The mucous glands, sebaceous structures and sebum could be seen under microscopy (10×)

Further laboratory examinations confirmed the diagnosis of vaginal paraganglioma. The immunohistochemical staining also showed SDHB (+), S100 (+), CgA (+), Syn (+), a low Ki-67 (with a proliferation index of 3 %), and AE1/AE3 (-). Please see Fig. 5 for further details. The rectal lesion had mucous glands and sebaceous structures under microscopy, drawing the diagnosis of mature SCT (see Fig. 6). As a matter of course, we also investigated 36 most common mutated genes related to PG through target sequencing; however, the results turned out to be negative (the genes were listed in supplement Table 1).

## Discussion and conclusions

As a rare tumor derived from the neural crest located in the paraganglion of the autonomic nervous system, PG accounts for 10-18 % of all chromaffin tumors [4]. According to their origins, PG can be classified as either parasympathetic or sympathetic. Those with parasympathetic characteristics mainly originate from the carotid body and the tympanic cavity of the head, neck and skull base. Most parasympathetic PG did not secrete catecholamine. Sympathetic PG on the other hand, originate from the sympathetic ganglia and can be located in any part of the body from the skull base to the bladder or prostate. Most sympathetic PG are functional.

Primary vaginal paraganglioma are extremely rare with only 10 previous cases reported [1, 5–13]. We have provided details extracted from the previous reports. Please see Table 1 for details. Compared with paraganglioma in other regions of the body, the median age of vaginal PG appears to be younger with a median of 26 years, ranging from 11 to 66. Vaginal PG can be located in all lateral walls of vagina, although it appears that they occur most commonly in the left or right lateral wall. The diameter ranges from 1.5 to 5.0 cm. According to the few cases reported, most of the tumors were non-functional or static, therefore most cases were identified through routine physical or gynecological examinations, or because of irregular vaginal bleeding. Unfortunately, only three definite functional cases have been reported, which causes diagnostic uncertainty before operation [6, 8, 12]. Many tumors may be misdiagnosed as vaginal leiomyoma or hemangioma because of abundant blood supply. Therefore, the final diagnosis often depends on postoperative pathological examination.

**Table 1:** The summarized table of all vaginal paraganglioma cases reported

Author	Year	Age(ys)	location	size	functional	symptoms	treatment	follow-up
Plate, W. P. [10]	1955	66	posterior vaginal wall	walnut size	nonfunctional	vaginal hemorrhage	surgery resection	not reported
Pezeshkpour, G. [11]	1981	22	anterior vaginal wall	3*2.5*1.5 cm	nonfunctional	asymptomatic	surgery resection	not reported
Parkes, S. E. [9]	1998	11	not reported	5 cm	nonfunctional	vaginal bleeding	surgery resection	not reported
Hassan, A. [8]	2003	24	left posterior vaginal fornix	2.5 cm	functional	hypertension, tachycardia and heart failure	surgery resection	4 months
Brustmann, H. [7]	2007	33	right lateral vaginal wall	two nodules, 1.9 and 1.4 cm	nonfunctional	vaginal bleeding	surgery resection	lost
Shen, J. G. [12]	2008	38	anterior vaginal wall	3.0 cm	functional	paroxysmal headaches, chest distress, palpitation	surgery resection	36 months
Akl, M. N. [13]	2010	65	vaginal apex	2.5 * 2.3*2 cm	nonfunctional	asymptomatic	artery embolism and surgery resection	not reported
Cai, T. [6]	2014	17	right vagina wall	3.5*3.0*2.5 cm	functional	vaginal bleeding	surgery resection	12 months
Sharma, S. [1]	2018	28	left lateral vaginal wall	3*3 cm	nonfunctional	asymptomatic	surgery resection	not reported
Wong, R. W. [5]	2020	15	left anterior vaginal wall	3 cm	nonfunctional	irregular heavy menses, dysmenorrhea, and anemia	surgery resection	56 months
Wang, Z.	2021	44	left vaginal wall	3.5 cm	functional	hypertension, palpitations and dizziness	surgery resection	under follow-up

Made up of endoderm, ectoderm, and mesoderm derivatives, primary teratoma mainly located in ovary, testis, anterior mediastinum, retroperitoneum, and sacrococcygeal area [14]. Adult sacrococcygeal teratoma (SCTs) is another rare tumor located behind the rectum and the embryological origin is still poorly understood. Although the classic theory regards germ cell from primitive yolk sac as the origination, others put it that the tumors may be caused by abnormal development of caudal cell mass or neural crest cells [15]. These lesions do not appear to manifest with symptoms and are therefore often found incidentally. The only common sign maybe that a patient experiences sacrococcygeal pain accompanied with constipation and frequent urination or urinary retention as the tumor progresses. In our case, the patient displayed typical symptoms of functional PG, including paroxysmal hypertension, palpitations and frequent headaches. Endocrine testing results suggested PG and radiographic images identified two possible lesions, one in vagina and the other in the rectum. Initially, we thought that both lesions were PG that led to the patient’s periodic symptoms; however, the results of pathological examinations identified vaginal paraganglioma as the culprit while the SCT may be just an incidental tumor.

Surgical resection is the standard treatment for vaginal paraganglioma and every effort should be made to completely remove the tumor. However, if the tumor’s volume is large, vaginal plasty should be considered. Vaginal paraganglioma appear to have quite favorable prognosis, and there is no report of tumor recurrence. However, we must note that this finding may be due to the limited cases and short follow-up periods, with the longest being 56 months [5]. In our case, the tumor volume was relatively large, which compressed the bladder vertically. In order to prevent bladder or ureteral injury during operation, we implanted bilateral ureteral stents pre-operatively.

In addition, in order to decrease the risk of postoperative rectal fistula, we consulted general surgeons before the initial operation and prepared for on-the-stage consultation during operation. Sufficient preoperative preparations can ensure the safety of patients and reduce the postoperative complications. For SCTs, surgical resection of retrorectal teratoma is generally considered the optimal method due to the risk of malignant transformation [2]. Therefore, laparoscopic SCT removal has become the modern method because it is less invasive, causing a smaller wound with quicker recovery [3]. In our case, we completely removed the lesion under laparoscopy without causing any damage to the rectum.

The mechanisms of tumorigenesis of vaginal PG are still poorly understood due to the limited number of cases. In 2019, Wong et al. identified the deletion of heterozygosity in exon 1 of SDHB gene as the etiology of a young girl’s vaginal PG [5]. However, in this case, we investigated 36 high-risk PG related genes including the SDH series but found no mutations. This suggested there was at least some heterogeneity in terms of the genes related to vaginal PG. The mechanisms and genes associated with SCT remain unknown, leaving huge space to be explored in the future.

In summary, we reported an extremely rare case of functional vaginal paraganglioma with adult sacrococcygeal teratoma. To the best of our knowledge, this is the first such case reported and therefore provides our experience and reflective thoughts. In order to identify related genetic mutations, we conducted target-sequencing but the result was negative. When comparing our findings with other similar instances we would suggest there is at least some heterogeneity in vaginal paraganglioma which demands further research. We could not determine whether the vaginal PG and SCT, were caused by the same etiology or whether this was purely incidental. We would encourage clinicians to record and report these peculiar cases to develop this evidence-base and to gain insight into the mechanisms involved.

## Supplementary Information

**Additional file 1.**

## Data Availability

To protect the patient’s privacy, data and materials including the sequencing data could be available with the permission from the corresponding author.

## Abbreviations

PG: Paraganglioma
^131^I-MIBG: Iodine-131 metaiodobenzylguanidine
NMN: Normetanephrine
NE: Norepinephrine
